# Supplementary material for: Loss of chromosome Y in regulatory T cells
Source: BMC Genomics. 2024 Mar 5;25:243. doi: 10.1186/s12864-024-10168-7 (PMC10913415; doi:10.1186/s12864-024-10168-7)
Supplement: Supplementary file 1 — Supplementary Material 1 [file 12864_2024_10168_MOESM1_ESM.pdf]

Supplementary Figures

Supplementary Figure 1

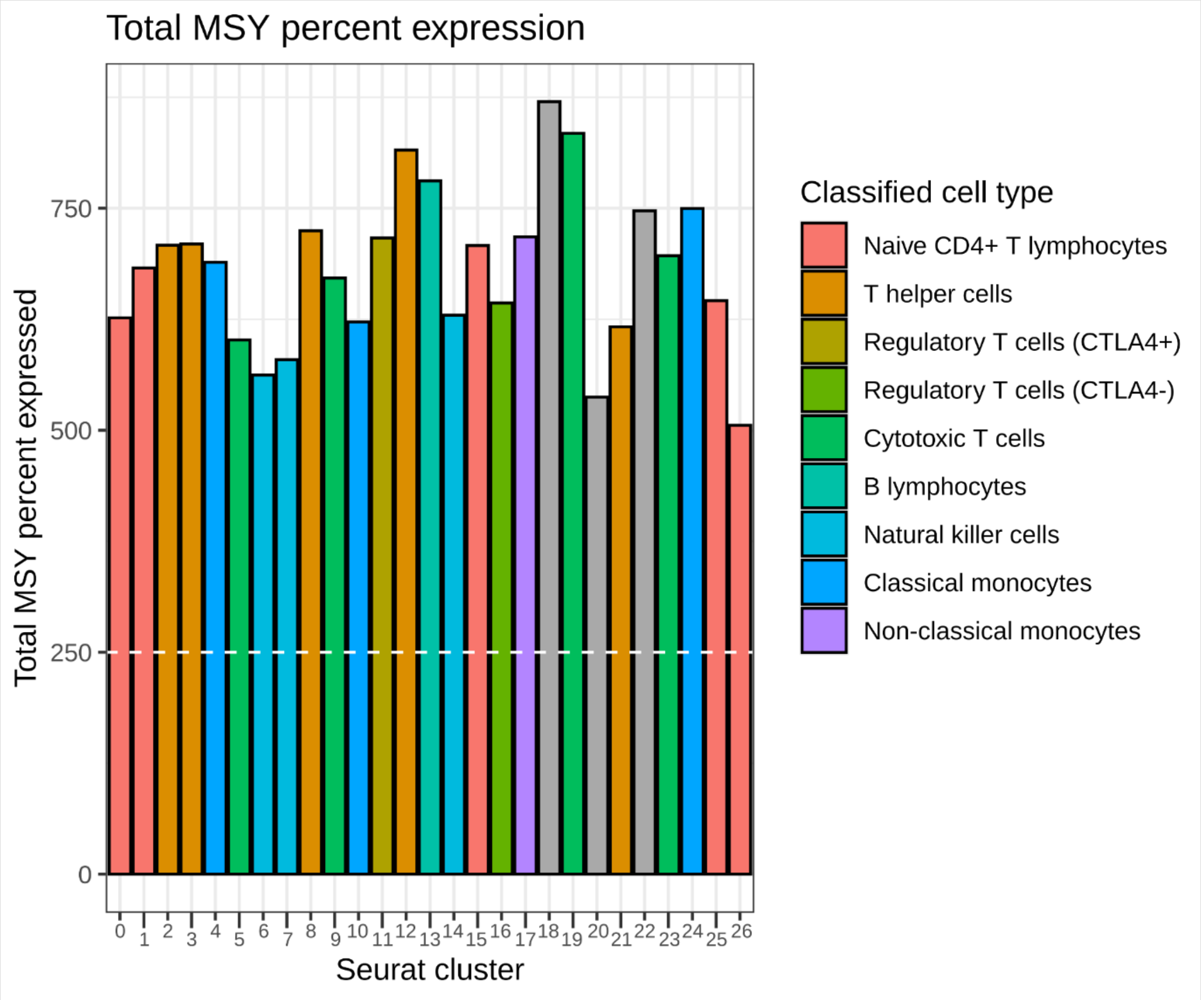

Supplementary Figure 1: Barplot showing the total cumulative MSY percent expression in each cluster, a method devised to qualify LOY assessment. The dashed line shows a cutoff at 250, the published QC threshold.

Supplementary Figure 2

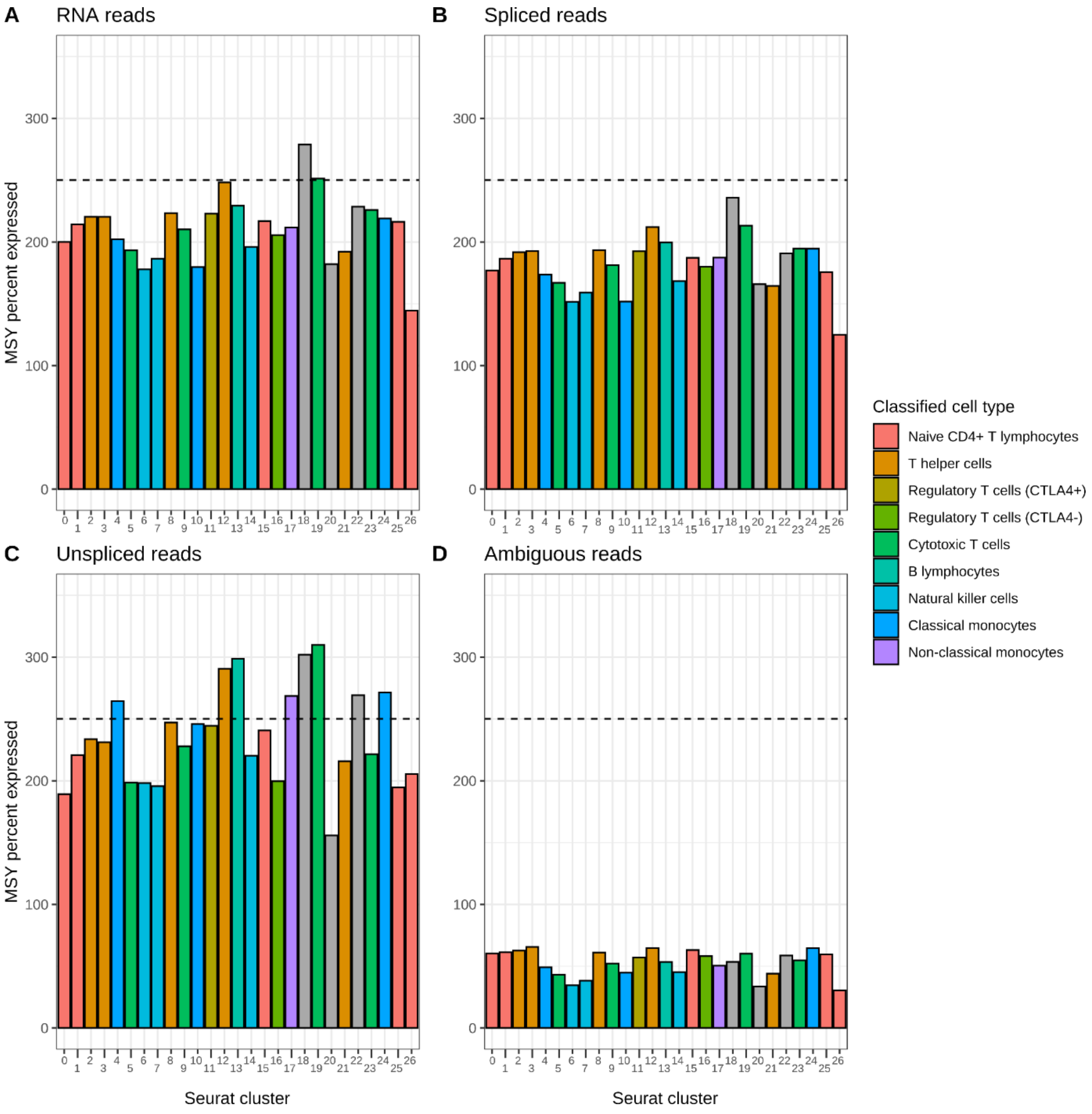

Supplementary Figure 2: The Barplot in *Supplementary Figure 1* separated by assay used to estimate LOY. Whereas the previous method of LOY classification only used the RNA reads identified by Cell Ranger (**A**), the new method considers all the different assays (**A**-**D**). The dashed line shows the published threshold required for LOY scoring.

Supplementary Figure 3

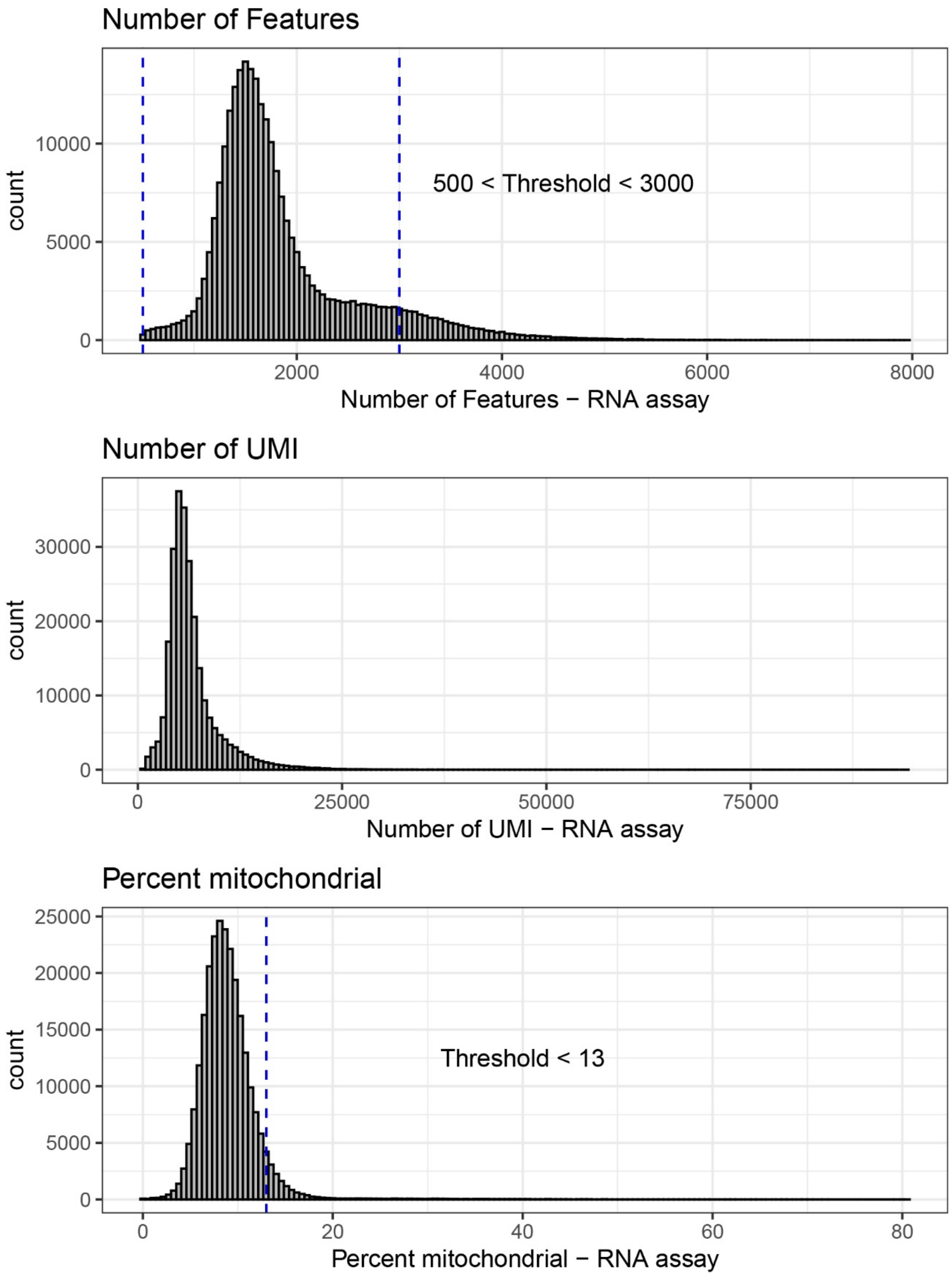

Supplementary Figure 3: Histograms presenting the filtering threshold used for the validation dataset. For each QC variable, the dashed line indicate the threshold applied.

Supplementary Figure 4

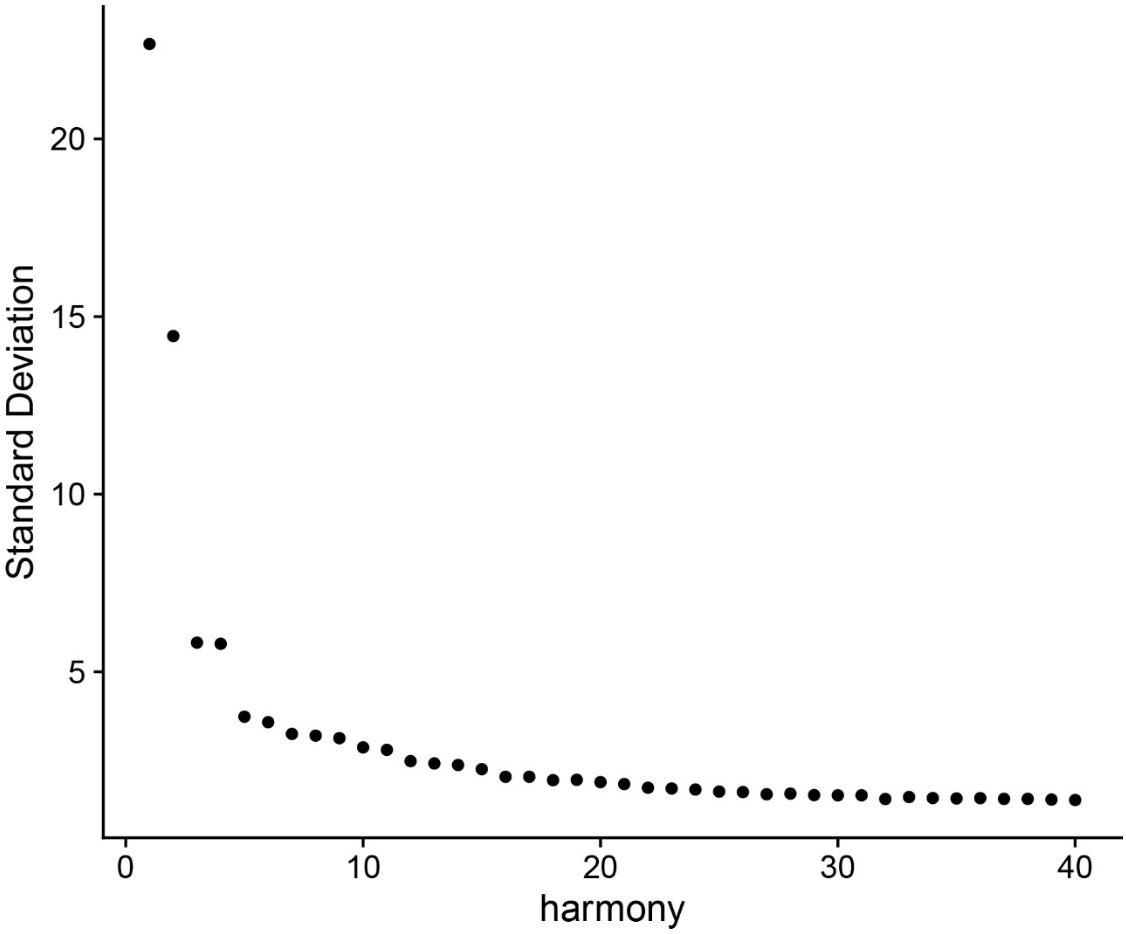

Supplementary Figure 4: Elbow plot used to pick the number of principle components used for clustering for the validation dataset. Based on this figure, the first 22 principle components were chosen.

Supplementary Figure 5

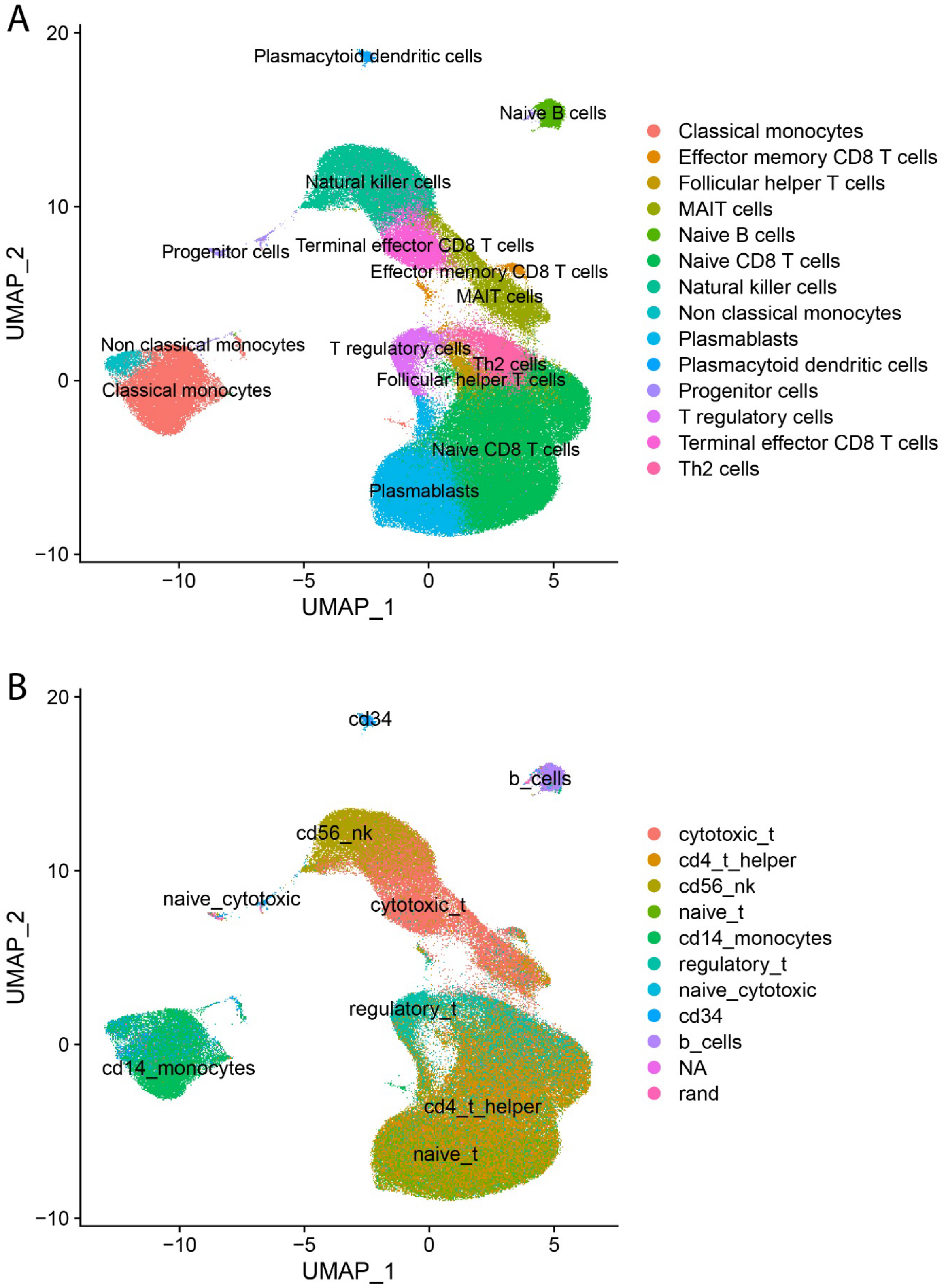

Supplementary Figure 5: UMAP showing the cell types predicted by the classification tools **A**) CIPR and **B**) SingleCellNet. This was used to guide the final cell type classification (*Supplementary Figure 10B*).

Supplementary Figure 6

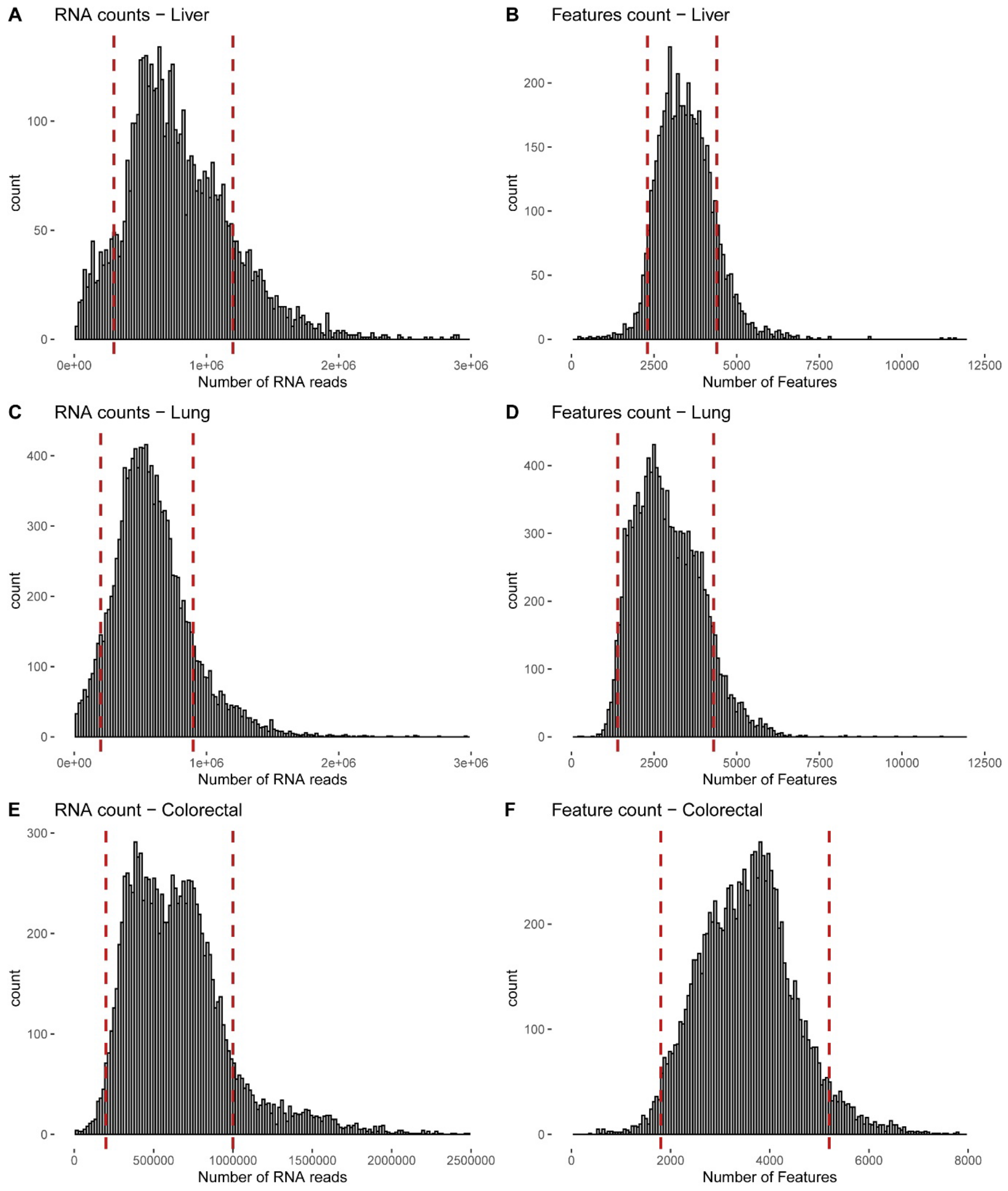

Supplementary Figure 6: Histograms presenting the filtering threshold used for the public datasets, similar to *Supplementary Figure 3*. Each dataset (here shown by row) was QC independently based on both RNA count and Feature count (by column).

Supplementary Figure 7

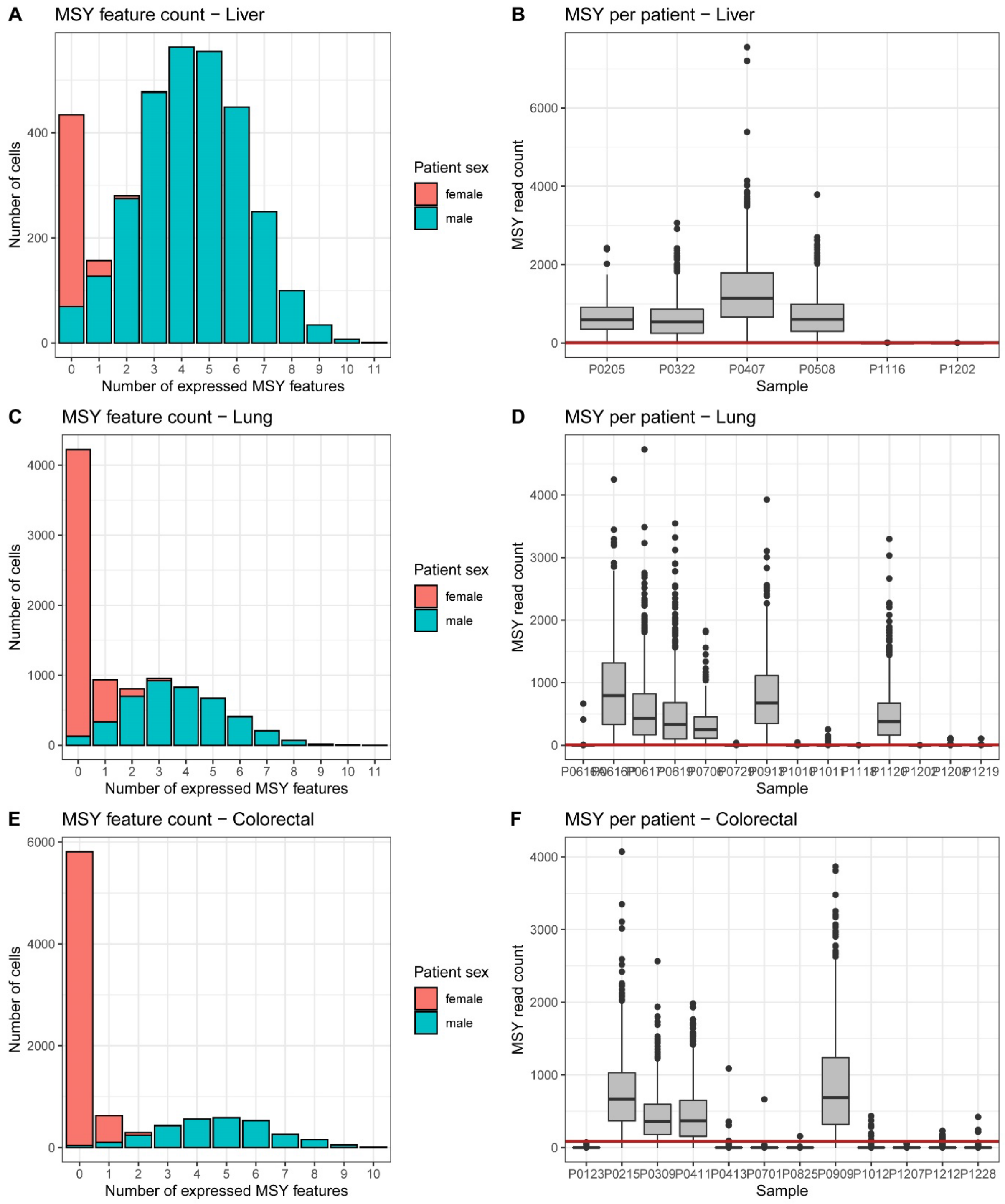

Supplementary Figure 7: Plots used to determine the method and threshold of LOY classification in the public dataset. This was done independently for each dataset, here shown by row.

Supplementary Figure 8

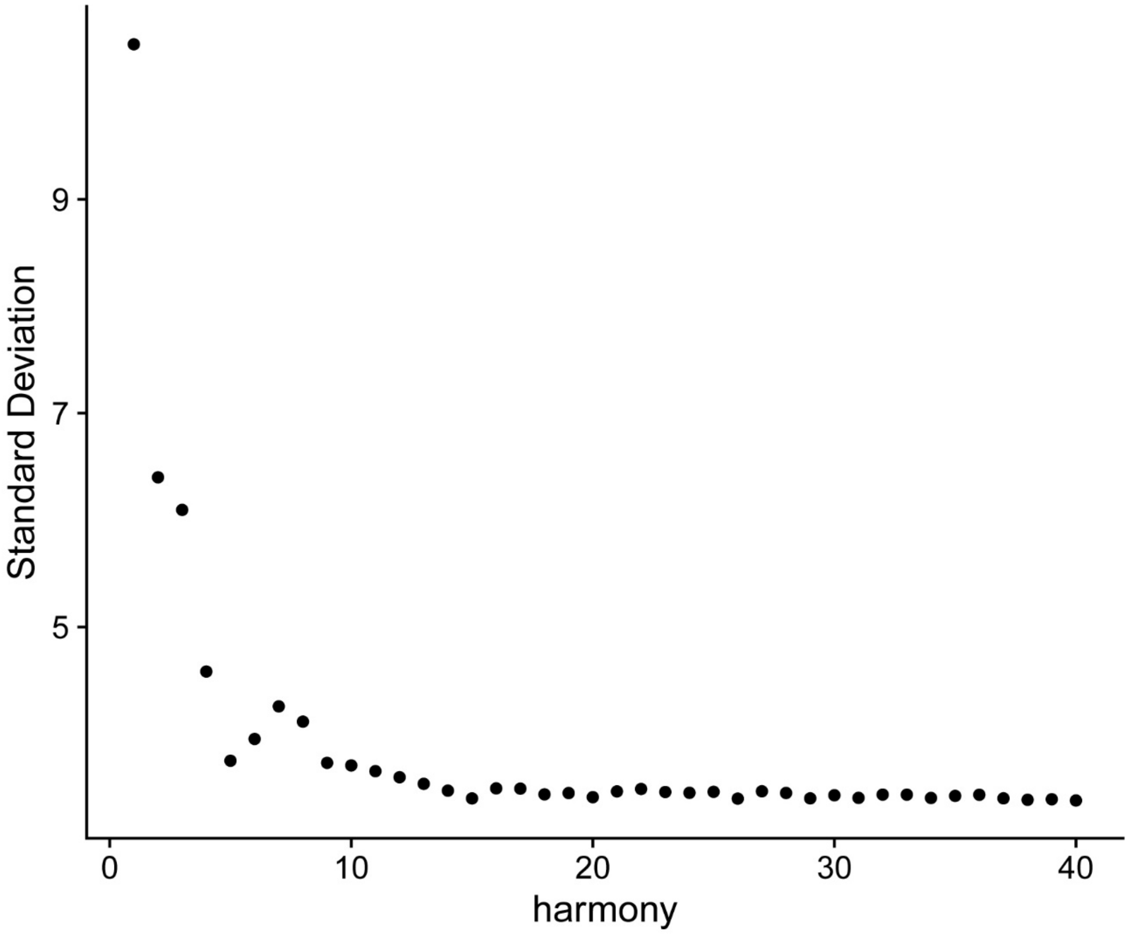

Supplementary Figure 8: Elbow plot used to pick the number of principle components used for clustering for the public datasets. Based on this figure, the first 15 principle components were chosen.

Supplementary Figure 9

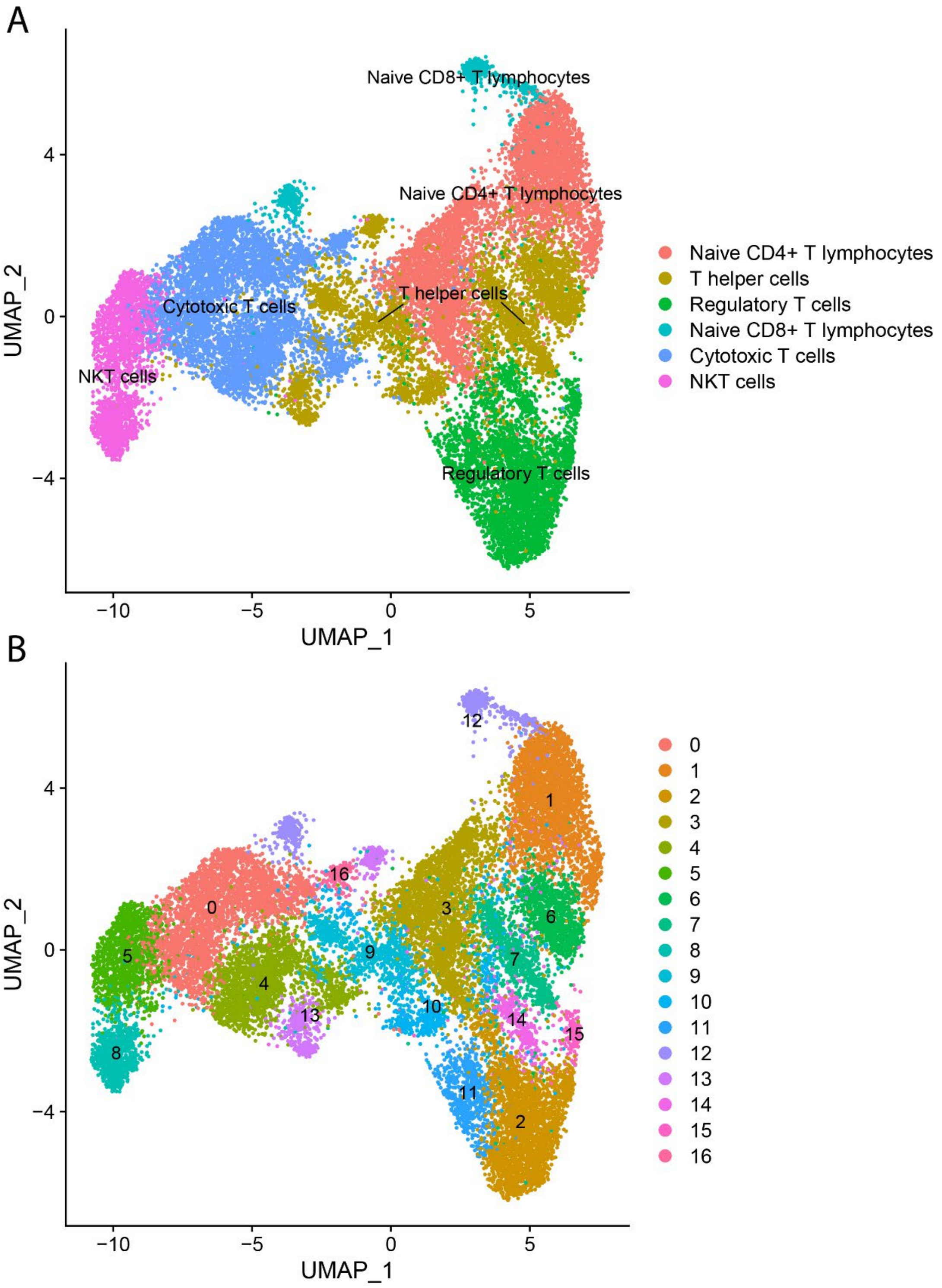

Supplementary Figure 9: UMAP from the public scRNAseq datasets, calculated on 15 principle components, showing **A**) the assigned cell types and **B**) clustering of cells with 0.6 resolution.

Supplementary Figure 10

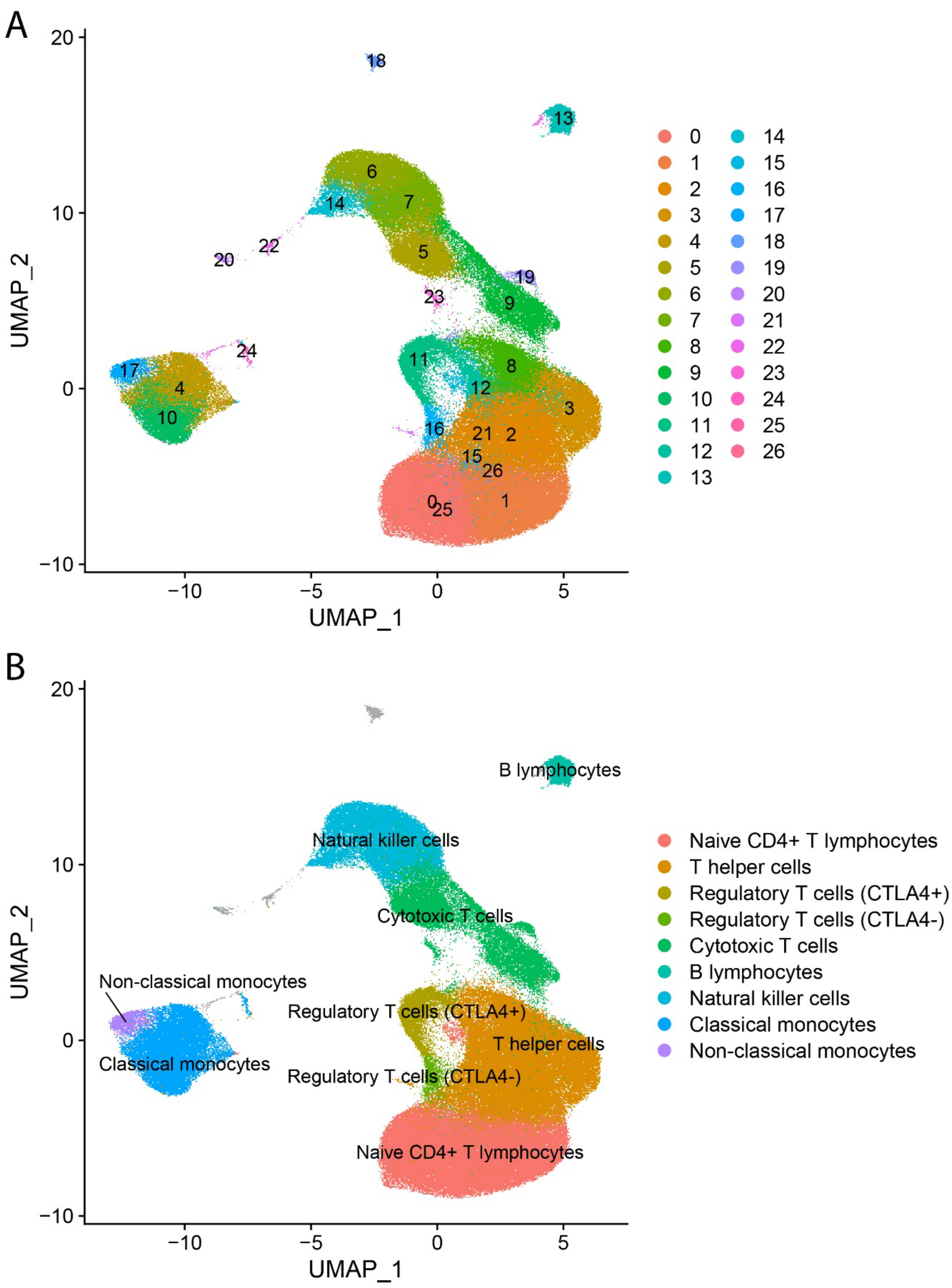

Supplementary Figure 10: UMAP from the validation scRNAseq datasets calculated on 22 principle components showing **A**) the clustering of cells with 0.9 resolution and **B**) the assigned cell types. Grey marks cells within clusters not identified as any of the studied cell types.

Supplementary Figure 11

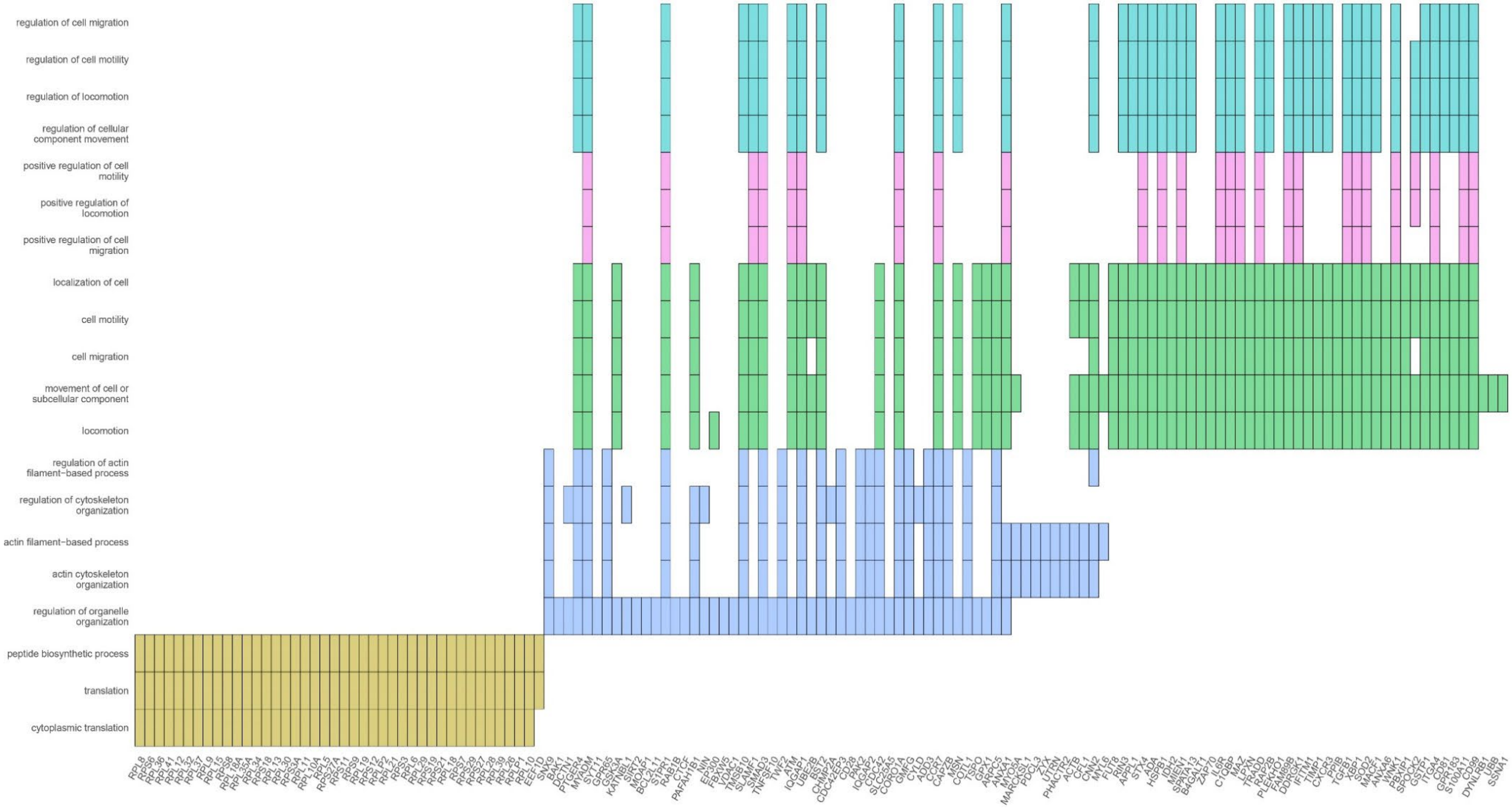

Supplementary figure 11: Inclusion of expressed genes in the different significant categories presented in *Figure 4*.

Supplementary Tables

Supplementary Table 1: Characteristics of participants.

Sample	Cohort	Age at sampling	Sample type sequenced
1	EpiHealth	75	CD4+ T lymphocyte Enriched
2	EpiHealth	73	CD4+ T lymphocyte Enriched
3	EpiHealth	81	CD4+ T lymphocyte Enriched
4	EpiHealth	74	CD4+ T lymphocyte Enriched
5	EpiHealth	76	CD4+ T lymphocyte Enriched
6	EpiHealth	77	CD4+ T lymphocyte Enriched
7	EpiHealth	77	CD4+ T lymphocyte Enriched
8	EpiHealth	71	CD4+ T lymphocyte Enriched
9	EpiHealth	78	CD4+ T lymphocyte Enriched
10	EpiHealth	71	CD4+ T lymphocyte Enriched
11	EpiHealth	75	CD4+ T lymphocyte Enriched
12	EpiHealth	71	CD4+ T lymphocyte Enriched
13	EpiHealth	78	CD4+ T lymphocyte Enriched
14	EpiHealth	73	CD4+ T lymphocyte Enriched
15	EpiHealth	78	CD4+ T lymphocyte Enriched
16	EpiHealth	78	CD4+ T lymphocyte Enriched
17	EpiHealth	76	CD4+ T lymphocyte Enriched
18	EpiHealth	68	CD4+ T lymphocyte Enriched
19	EpiHealth	77	CD4+ T lymphocyte Enriched
20	EpiHealth	80	CD4+ T lymphocyte Enriched
21	EpiHealth	54	CD4+ T lymphocyte Enriched
22	EpiHealth	78	CD4+ T lymphocyte Enriched
23	EpiHealth	78	CD4+ T lymphocyte Enriched
24	EpiHealth	75	CD4+ T lymphocyte Enriched
25	EpiHealth	68	CD4+ T lymphocyte Enriched
26	EpiHealth	73	CD4+ T lymphocyte Enriched
27	EpiHealth	80	CD4+ T lymphocyte Enriched
28	EpiHealth	80	CD4+ T lymphocyte Enriched
29	EpiHealth	78	CD4+ T lymphocyte Enriched
30	EpiHealth	75	CD4+ T lymphocyte Enriched
31	UCAN	77	CD4+ T lymphocyte Enriched
32	UCAN	78	CD4+ T lymphocyte Enriched
33	UAD	80	PBMCs
34	UAD	74	PBMCs
35	UAD	66	PBMCs
36	UAD	83	PBMCs
37	UAD	72	PBMCs
38	UAD	65	PBMCs
39	UAD	57	PBMCs
40	UAD	86	PBMCs
41	UAD	82	PBMCs
42	UAD	71	PBMCs
43	UAD	89	PBMCs
44	UAD	73	PBMCs
45	UAD	79	PBMCs
46	UAD	84	PBMCs
47	UAD	85	PBMCs
48	UAD	81	PBMCs
49	UAD	68	PBMCs
50	UAD	83	PBMCs
51	UAD	70	PBMCs
52	UAD	77	PBMCs
53	UAD	77	PBMCs
54	UAD	94	PBMCs
55	UAD	78	PBMCs

Supplementary Table 2: Statistical model to test whether the number of cells in each cell type and sample depends on LOY. Type III ANOVA based on a negative binomial model. Logarithmic link function. Cell count was used as response variable. Deviance of 89.10299 on 58 residuals.

	LR Chisq	Df	Pr(>Chisq)
scale(percent.LOY)	6.591	1	0.01025 *
cell.type	81.317	5	4.45e-16 ***
cancer.type	2.391	2	0.30263
scale(percent.LOY):cell.type	3.394	5	0.63941
cell.type:cancer.type	14.213	10	0.16348
scale(percent.LOY):cancer.type	8.423	2	0.01483 *

Supplementary Table 3: Statistical model to test whether proportion of LOY cells is significantly different between tissues. Type III ANOVA based on a quasibinomial model. Logistic link function. Proportion of LOY cells compared with normal cells used as response variable. Deviance of 335.8531 on 199 residuals.

	LR Chisq	Df	Pr(>Chisq)
cell.type	64.094	5	1.727e-12 ***
tissue	8.426	2	0.014804 *
cancer.type	13.436	2	0.001209 **
tissue:cancer.type	16.326	4	0.002611 **
cell.type:cancer.type	22.680	10	0.011990 *

Supplementary Table 4: Statistical model to test whether the proportion of cells in each cell type and sample depends on LOY. Type III ANOVA based on a quasibinomial model. Logistic link function. Proportion of cells in current cell type compared with other cell types used as response variable. Deviance of 38355.77 on 616 residuals. Batch denotes sample type, either CD4+ T lymphocyte enrichment or PBMC.

	LR Chisq	Df	Pr(>Chisq)
scale(percent.LOY)	5.63	1	0.0176167 *
cell.type	810.08	11	< 2.2e-16 ***
batch	14.85	1	0.0001166 ***
scale(percent.LOY):cell.type	22.73	11	0.0192874 *
scale(percent.LOY):batch	1.75	1	0.1864470
cell.type:batch	617.53	11	< 2.2e-16 ***

Supplementary Table 7: The results from the GSEA. Only gene sets significant after adjusting for multiple testing (Benjamini-Hochberg) was included.

ID	Description	setSize	enrichmentScore	NES	pvalue	p.adjust
GO:0002181	cytoplasmic translation	122	0.64020	3.02877	1.00E-10	4.31E-08
GO:0006412	translation	402	0.31803	1.66259	1.00E-10	4.31E-08
GO:0040011	locomotion	396	-0.50748	-1.66080	1.00E-10	4.31E-08
GO:0040012	regulation of locomotion	241	-0.55807	-1.80073	1.00E-10	4.31E-08
GO:0043043	peptide biosynthetic process	406	0.31457	1.70744	1.00E-10	4.31E-08
GO:0048870	cell motility	365	-0.51881	-1.69465	1.00E-10	4.31E-08
GO:0051674	localization of cell	365	-0.51881	-1.69465	1.00E-10	4.31E-08
GO:2000145	regulation of cell motility	233	-0.55904	-1.80180	1.00E-10	4.31E-08
GO:0006928	movement of cell or subcellular component	456	-0.49124	-1.61246	1.36E-10	5.22E-08
GO:0030334	regulation of cell migration	222	-0.56354	-1.81302	1.86E-10	6.23E-08
GO:0016477	cell migration	336	-0.51802	-1.68870	1.99E-10	6.23E-08
GO:0051270	regulation of cellular component movement	254	-0.53733	-1.73692	4.48E-10	1.29E-07
GO:0051493	regulation of cytoskeleton organization	176	-0.57902	-1.84455	5.70E-10	1.51E-07
GO:0030036	actin cytoskeleton organization	191	-0.56148	-1.79487	1.88E-09	4.63E-07
GO:0030029	actin filament-based process	207	-0.55552	-1.78211	4.22E-09	9.71E-07
GO:0030335	positive regulation of cell migration	136	-0.59348	-1.86614	6.09E-09	1.28E-06
GO:0040017	positive regulation of locomotion	143	-0.59294	-1.86954	6.34E-09	1.28E-06
GO:0032970	regulation of actin filament-based process	118	-0.62183	-1.93776	7.70E-09	1.47E-06
GO:0033043	regulation of organelle organization	409	-0.47702	-1.56211	1.27E-08	2.31E-06
GO:2000147	positive regulation of cell motility	141	-0.59225	-1.86585	1.35E-08	2.33E-06
GO:0007015	actin filament organization	127	-0.59352	-1.85858	1.91E-08	3.14E-06
GO:0044087	regulation of cellular component biogenesis	313	-0.49691	-1.61671	2.09E-08	3.28E-06
GO:0051272	positive regulation of cellular component movement	145	-0.57704	-1.82083	3.93E-08	5.89E-06
GO:0032956	regulation of actin cytoskeleton organization	108	-0.61788	-1.91404	4.14E-08	5.94E-06
GO:0051128	regulation of cellular component organization	703	-0.42887	-1.41855	8.23E-08	1.14E-05
GO:0019886	antigen processing and presentation of exogenous peptide antigen via MHC class II	14	0.87773	2.59650	8.57E-08	1.14E-05
GO:0051130	positive regulation of cellular component organization	339	-0.48565	-1.58351	1.40E-07	1.79E-05
GO:0007010	cytoskeleton organization	381	-0.46347	-1.51540	4.73E-07	5.70E-05
GO:0110053	regulation of actin filament organization	91	-0.62552	-1.91148	4.79E-07	5.70E-05
GO:0044089	positive regulation of cellular component biogenesis	176	-0.53450	-1.70273	5.01E-07	5.76E-05
GO:0032879	regulation of localization	740	-0.42002	-1.39041	6.43E-07	7.15E-05
GO:0002501	peptide antigen assembly with MHC protein complex	10	0.91701	2.44862	8.68E-07	9.35E-05
GO:0019884	antigen processing and presentation of exogenous antigen	24	0.74652	2.55763	1.13E-06	1.18E-04
GO:0050900	leukocyte migration	102	-0.58435	-1.80237	1.47E-06	1.49E-04
GO:0002504	antigen processing and presentation of peptide or polysaccharide antigen via MHC class II	16	0.82332	2.53292	1.58E-06	1.56E-04
GO:0002478	antigen processing and presentation of exogenous peptide antigen	20	0.75876	2.48296	3.19E-06	3.05E-04
GO:0008154	actin polymerization or depolymerization	79	-0.61307	-1.84898	4.00E-06	3.73E-04
GO:0022604	regulation of cell morphogenesis	86	-0.60384	-1.83605	4.19E-06	3.80E-04
GO:0007166	cell surface receptor signaling pathway	656	-0.41407	-1.36819	4.39E-06	3.88E-04
GO:0002495	antigen processing and presentation of peptide antigen via MHC class II	15	0.82598	2.49269	6.68E-06	5.76E-04
GO:0071674	mononuclear cell migration	54	-0.66636	-1.93019	1.14E-05	9.60E-04
